# Genetic Structure and Molecular Diversity of Cacao Plants Established as Local Varieties for More than Two Centuries: The Genetic History of Cacao Plantations in Bahia, Brazil

**DOI:** 10.1371/journal.pone.0145276

**Published:** 2015-12-16

**Authors:** Elisa S. L. Santos, Carlos Bernard M. Cerqueira-Silva, Gustavo M. Mori, Dário Ahnert, Durval L. N. Mello, José Luis Pires, Ronan X. Corrêa, Anete P. de Souza

**Affiliations:** 1 Laboratory of Applied Molecular Genetics, Department of Exact and Natural Sciences, State University of Southwest Bahia, Itapetinga, Bahia, Brazil; 2 Center for Molecular Biology and Genetic Engineering, University of Campinas, Campinas, São Paulo, Brazil; 3 Genetics and Biotechnology Center, Department of Biological Science, State University of Santa Cruz, Ilhéus, Bahia, Brazil; 4 Bahian Federal Institute of Education Science and Technology, Uruçuca, Bahia, Brazil; 5 Cocoa Research Center, Executive Committee of the Plan for Cacao Farming, Itabuna, Bahia, Brazil; 6 Department of Plant Biology, Biology Institute, University of Campinas (UNICAMP), Campinas, São Paulo, Brazil; National Institute of Plant Genome Research (NIPGR), INDIA

## Abstract

Bahia is the most important cacao-producing state in Brazil, which is currently the sixth-largest country worldwide to produce cacao seeds. In the eighteenth century, the Comum, Pará and Maranhão varieties of cacao were introduced into southern Bahia, and their descendants, which are called ‘Bahian cacao’ or local Bahian varieties, have been cultivated for over 200 years. Comum plants have been used to start plantations in African countries and extended as far as countries in South Asia and Oceania. In Brazil, two sets of clones selected from Bahian varieties and their mutants, the Agronomic Institute of East (SIAL) and Bahian Cacao Institute (SIC) series, represent the diversity of Bahian cacao in germplasm banks. Because the genetic diversity of Bahian varieties, which is essential for breeding programs, remains unknown, the objective of this work was to assess the genetic structure and diversity of local Bahian varieties collected from farms and germplasm banks. To this end, 30 simple sequence repeat (SSR) markers were used to genotype 279 cacao plants from germplasm and local farms. The results facilitated the identification of 219 cacao plants of Bahian origin, and 51 of these were SIAL or SIC clones. Bahian cacao showed low genetic diversity. It could be verified that SIC and SIAL clones do not represent the true diversity of Bahian cacao, with the greatest amount of diversity found in cacao trees on the farms. Thus, a core collection to aid in prioritizing the plants to be sampled for Bahian cacao diversity is suggested. These results provide information that can be used to conserve Bahian cacao plants and applied in breeding programs to obtain more productive Bahian cacao with superior quality and tolerance to major diseases in tropical cacao plantations worldwide.

## Introduction

Brazil is the most important cacao-producing country in the Americas, with 253,211 tons of cacao beans produced in 2012 (http://faostat3.fao.org). Sixty-two percent of Brazilian cacao production occurs in the southern part of Bahia, which encompasses approximately 540,000 ha and yields an average of 300 kg/ha [1]. In southern Bahia, approximately 70% of cacao cultivation occurs in cabruca agroforestry systems [2], which are characterized by understory tree species in Atlantic Forests. This system has permitted the conservation of native flora and fauna [3,4]. In this region, the average temperature ranges from 20 to 27°C, and annual rainfall may reach 2,350 mm (http://www.inpe.br/). Because of the abundant rainfall, cacao is produced for approximately nine months per year, and the production season is divided into two harvest periods: the main period from November to February and secondary period (known locally as temporão) from May to September.

The first introduction of cacao into southern Bahia occurred in 1746 when seeds of the Amelonado variety were imported from the Amazonian Region by a Frenchman and planted in the municipality of Canavieiras on the banks of the River Pardo [5]. The specific place from where the cacao seeds were obtained is unclear, but previous reports indicate Pará, another Brazil State, as a probable region [5]. This initial introduction was the origin of the ‘Comum’ variety, and over the course of two centuries, the descendants of these plants spread and established nearly all of the Bahian cacao plantations as well as commercial plantations in Espírito Santo State. Bahian cacao was also used to establish cacao plantations in African countries beginning in 1822, and its important germplasm propelled certain West Africa countries to become the main cacao producers worldwide in the early twentieth century [6,7].

In Bahia, additional introductions (also from Pará State) between 1874 and 1876 brought the ‘Maranhão’ and ‘Pará’ varieties, which differ from each other as well as from the Comum variety, primarily in their fruit characteristics [5,8]. Spontaneous mutants occurred in these cacao founders, generating varieties such as Almeida and Catongo, which produce white seeds [9]. Because of the unique characteristics of these varieties, they were naturalized as Bahian cacao or Bahia local cacao cultivars, and the state is currently considered a secondary area of cacao diversity [8].

In the 1930s and 1940s, the first selective breeding of Bahian varieties was performed in farmers’ fields in Bahia to obtain more productive clones, and it resulted in the SIC and SIAL series (Cacao Institute Selection and East Agronomic Institute Selection, respectively) in Bahia and EEG (Selection of the Goitacazes Experimental Station) clones in Espírito Santo [10]. These clones are considered representative of the genetic material that was introduced and disseminated throughout the Bahian cacao plantations [11,12].

The initial characterization of the cacao varieties planted on farms (Comum, Pará and Maranhão) and SIC and SIAL clones indicated good productivity and variability for certain traits, such as fruit diameter and seed weight [5,11]. These and other evaluations were specifically designed to assess the productivity and unique characteristics of Bahian cacao plants [5,13,14] and performed to identify the best clones for use in the cacao genetic breeding program of the Cocoa Research Center (CEPEC) of the Executive Committee of the Plan for Cacao Farming (CEPLAC).

The SIC and SIAL clones as well as clones introduced from other countries were used in breeding programs to generate hybrid cultivars with high productivity that are resistance to pod rot (until 1989, the most common cacao disease in Bahia) and have good-quality beans for industry [9]. However, with the outbreak of witches’ broom disease (caused by the fungus *Moniliophthora perniciosa*) in Bahia in 1989, the development of hybrid cultivars stopped, and the selection and breeding of resistant clonal cultivars occurred through recurrent selection or resistant plant selection from agricultural fields [15]. Indeed, prior to the witches’ broom outbreak, Comum, Pará and Maranhão were the predominant cacao varieties in Bahia, and until at least 2003, they accounted for approximately 50% of the land cultivated for cacao in the state [13]. The decline in Bahia's global rank from second to sixth in cacao production highlights the consequences of this disease on Bahia’s economy and justifies the replacement of a number of the originally introduced plant varieties with those that are more disease resistant. Additionally, the low prices associated with the cacao market as well as specific environmental factors contributed to the cacao crisis in Bahia [16].

Currently, only a limited number of farms, which are largely smallholdings, in southern Bahia exclusively plant Bahian varieties. A number of these plantations consist of 'historical trees,' or plants that have been maintained by the farmer’s family for at least two generations and are still classified as productive. To overcome the cacao production crisis caused by witches’ broom, certain Bahian farmers have invested in the gourmet chocolate market, in which flavorful cacao is worth three times the price of standard cacao. The Maranhão and Catongo (a spontaneous mutant of Comum cacao) varieties are currently used in the fine cacao market, and they have been reported to produce less astringent and more flavorful chocolate [17].

Because of the history of Bahian cacao’s introduction and spread as well as its importance as an adapted genetic resource in Bahia and other cacao producing areas, the molecular characterization of Bahian cacao is important to elucidate its genetic and structural diversity and identify plants that are optimal for use in breeding programs. Thus, this study aimed to describe the genetic diversity and structure of local Bahian cacao cultivars from farms and germplasm banks using microsatellite markers. We have discussed the effective representativeness of the Bahian cacao in germplasm banks, effects of witches’ broom on the structure and diversity of cacao in Bahia and perspectives on the use of Bahian cacao.

## Materials and Methods

### Plant materials and DNA isolation

**Fig 1:**
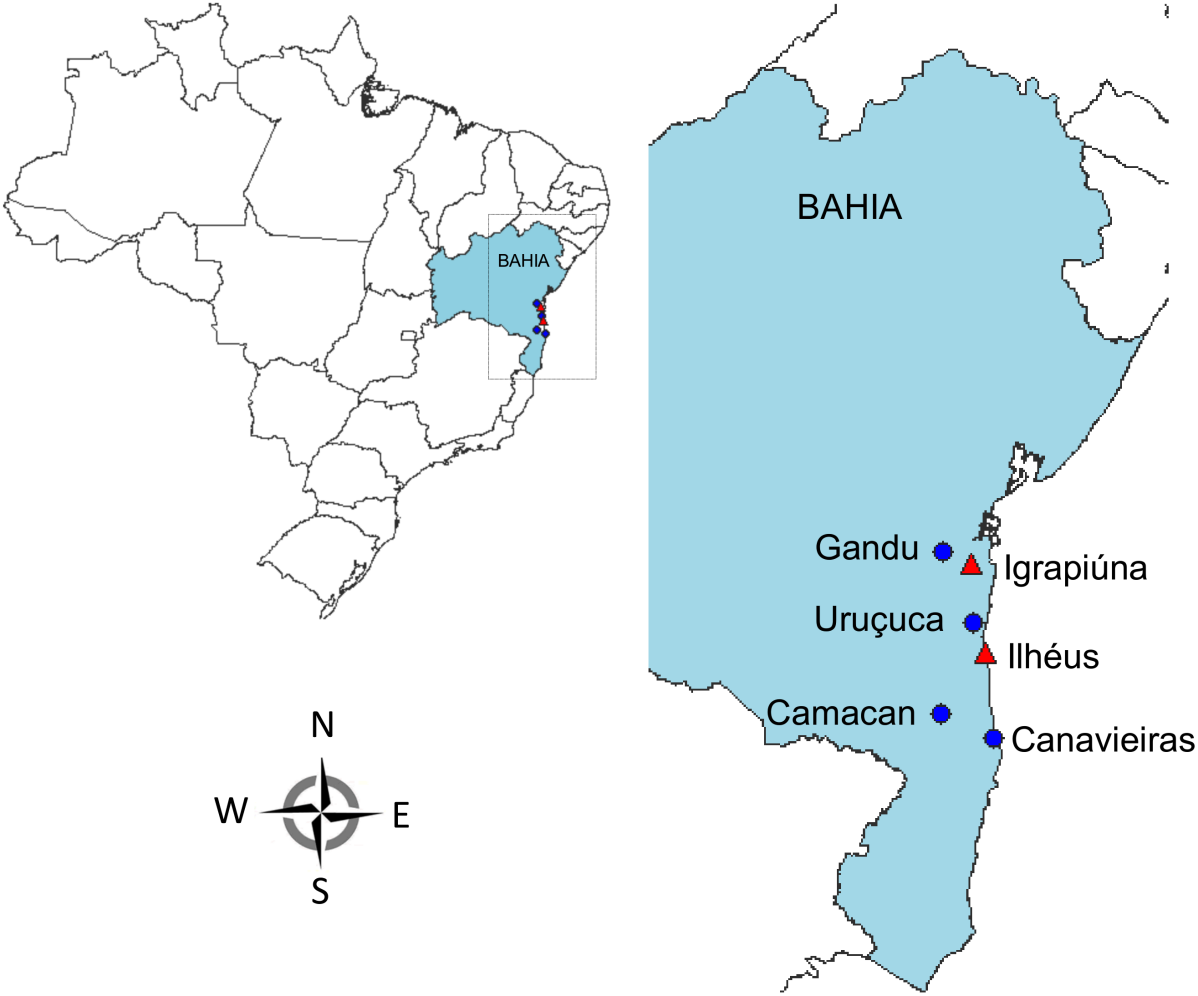
Illustrative map of the Bahian areas where cacao samples were obtained. Map built using speciesMapper, a free tool of the software *species*Link (http://splink.cria.org.br). The southeast region of Bahia is highlighted and includes the municipalities where the cacao leaves were collected. Blue circles indicate the municipalities of collection of the genotypes; red triangles indicate the municipalities of collection of the clones (Cocoa Research Center in the Ilhéus Municipality and Juliana's Valley Farm in the Igrapiúna Municipality).

The leaves of the 279 cacao trees used in this study were obtained from farms and educational and research institutions in six municipalities of southern Bahia, Brazil (Fig 1). Hereafter, the plants of seminal origin are referred to as genotypes and those generated from grafts or cuttings are referred to as clones.

**Fig 2:**
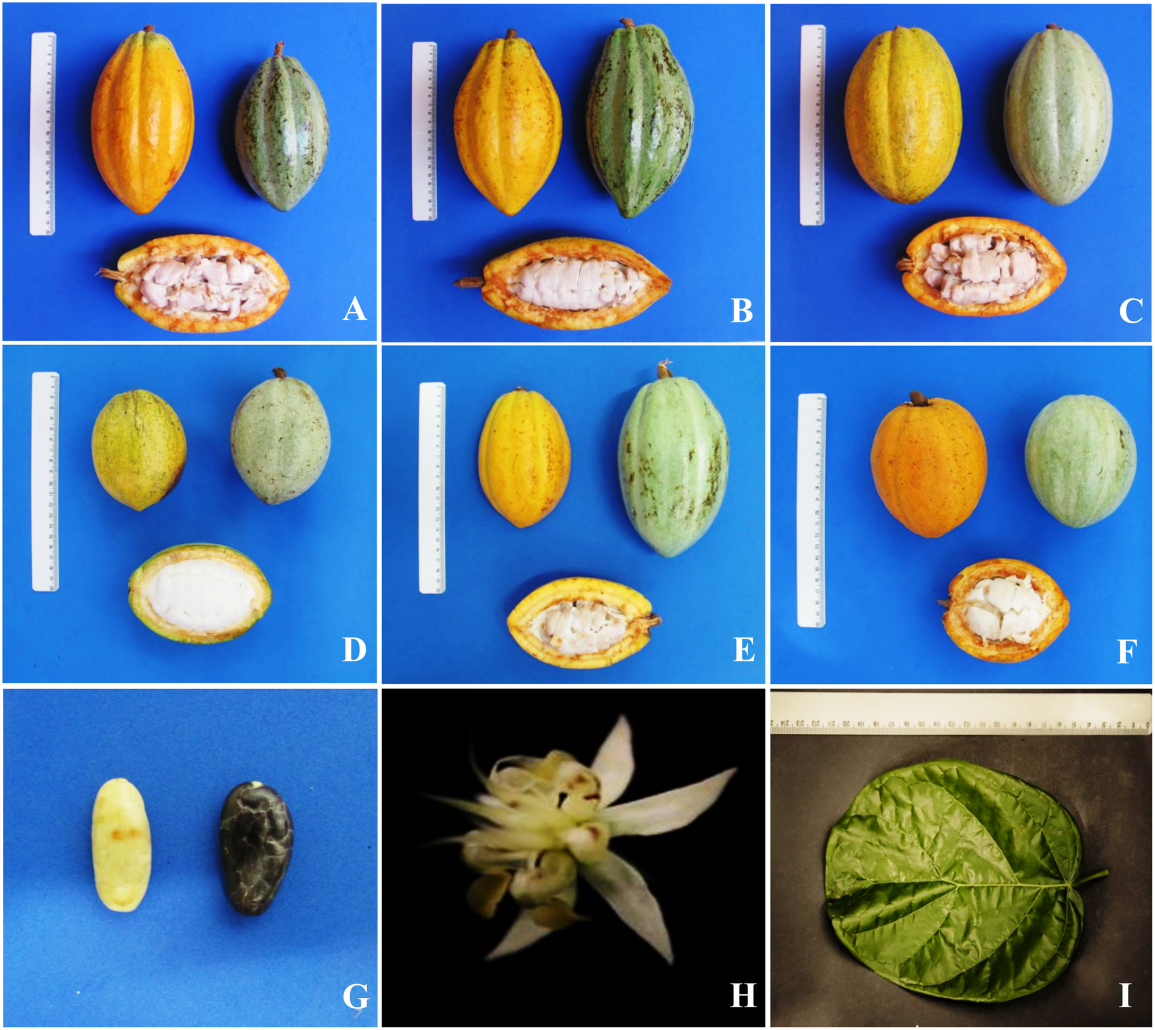
Bahian cacao varieties. A to C varieties introduced into southern Bahia in the 18^th^ century—(A) Comum; (B) Maranhão and (C) Pará. D to I mutants of Bahian cacao—(D) Almeida and (E) Catongo (mutants of Comum variety that present white leaves, flowers and seeds) and (F) Laranja (mutant of Pará variety). (G) and (H) seeds and flower of Catongo, respectively. (I) leaf of Jaca cacao.

The genotypes and clones used in this work and their locations of origin are listed in S2 and S3 Tables, respectively. A total of 176 genotypes were obtained from seven farms and two research institutions, the Bahian Federal Institute of Education Science and Technology (IFBAIANO) and CEPLAC, located in four municipalities of southern Bahia: Canavieiras, Camacan, Gandu and Uruçuca. The plants were randomly chosen from those identified as varieties of Bahian cacao, and the identification was based on information provided by the farmers as well as on the fruit characteristics of the three main types of Bahian cacao: Comum, Pará and Maranhão (Fig 2 and S1 Table). Because hybridization occurs among the plants over time, accurately differentiating Bahian varieties using botanical characteristics is not always possible because the fruits share characteristics of two or more types. Bahian cacao mutants were also analyzed, including one ‘jaca’ cacao genotype and fifteen ‘laranja’ cacao genotypes (Portuguese terms for jackfruit and orange, respectively). Jaca cacao plants have small rounded leaves (Fig 2) that are similar to those of jackfruit trees [8], three plagiotropic branches instead of the five commonly observed in cacao, and abnormalities in flower formation [18]. Laranja cacao is a subvariety of Pará that is characterized by small rounded fruits similar to orange fruits (Fig 2).

Additionally, 103 plants were sampled from the clones in the Cocoa Research Center (CEPEC/CEPLAC) in the Ilhéus Municipality and from the Juliana's Valley Farm in the Igrapiúna Municipality. Fifty-one plants were clones and represented the first cacao selections in Bahia; these plants are descendants of the plants that founded the commercial regions in the state. Of these clones, 31 were selected by the former Cacao Institute of Bahia (currently IFBAIANO) in the 1930s and are designated SIC clones and 20 were selected by the now-defunct Agronomic Institute of East in the 1940s and are designated SIAL clones. An additional 52 clones from different geographic regions (S3 Table) were included and are hereafter denoted as reference clones. Among these clones, eleven are clonal cultivars (also known as ‘modern cultivars’), and they have been planted on many Bahian farms.

Young leaves from each genotype and clone were collected, and DNA was obtained using the Cetyl Trimethyl Ammonium Bromide method according to Faleiro et al. [19].

### SSR markers, PCR and electrophoresis

Thirty microsatellite markers were used to genotype the plants (S4 Table), including 27 genomic markers developed by Santos et al. [20] and Lanaud et al. [21], and 3 functional markers developed by Lima et al. [22]. The polymerase chain reaction (PCR) analysis was performed in a final volume of 20 μL containing 6 ng DNA template, 1× PCR buffer (20 mM Tris-HCl, pH 8.4, 50 mM KCl), 1.5 mM MgCl_2_, 0.2 μM of each dNTPs, 0.5 μM of each forward and reverse primers and 1 U of Taq DNA polymerase (Invitrogen, Carlsbad, CA, USA). The amplification reactions for each locus were conducted according to the protocols described by the authors of the respective articles referenced above. The amplified products were resolved using vertical electrophoresis on 1× TBE/6% denaturing polyacrylamide gels at 75 W for approximately 2 h and then stained with silver nitrate according Creste et al. [23]. For each run, eight DNA samples were repeated as controls. The product sizes were determined by comparison with a 10-bp DNA ladder (Invitrogen).

### Analyses of population structure

To assess genetic diversity among the plants, three different approaches were used: a Bayesian model-based approach, Nei’s G_ST_ statistic and principal coordinate analysis (PCoA). In the first approach, the genetic structure was inferred using STRUCTURE software v. 2.3.4 [24]. The admixture ancestry model was implemented, and the burn-in periods and replications were 100,000 and 400,000 iterations, respectively. Two STRUCTURE runs were performed using the above parameters, with one run including all 279 plants (complete dataset) and the additional run only using the plants that were classified in the first run as part of the Bahian cacao group. In the STRUCTURE analysis of the total data, the number of tested clusters (K) ranged from 1 to 10, and 20 independent runs were performed for each K tested. For the run with only the Bahian cacao data, the K value ranged from one to six.

Structure Harvester software v. 0.6.93 [25] was used to analyze the Structure output, and the optimal K values were calculated using Evanno’s ΔK *ad hoc* statistics [26]. The best alignment over the 20 runs for the optimal K values was obtained using the Cluster Matching and Permutation Program v. 1.1.2 (Clumpp) [27], and the results were visualized using DISTRUCT software [28].

The levels of structure estimated from the pairwise G_ST_ values were obtained using 999 permutations and 1,000 bootstraps in GenAlEx software v. 6.5 [29]. Using this approach, plants sampled from farms and germplasm banks (SIC and SIAL) and modern cultivars currently recommended for planting in Bahia were considered as distinct groups. The dispersion of the total data in a bidimensional diagram was determined using PCoA, and distance and projection were calculated using GenAlEx software v. 6.5.

### Genetic diversity analyses

Descriptive statistics for the genotyping data were generated using GenAlEx v. 6.5 to estimate the total number of alleles, allelic frequencies at each locus and observed and expected heterozygosities. These parameters were determined for the total data, which included 279 genotypes, and applied to the independent measurements performed for the groups and subgroups delimited by the Structure software. The plants sampled at different times in Bahia were accounted for, and the modern cultivars were treated as a subgroup in the analysis.

For the total data, the polymorphism levels at each locus, polymorphism information content (PIC), Hardy-Weinberg equilibrium deviations per locus and fixation index (F) per locus and for all loci were assessed using GenAlEx v. 6.5. This software was also used to calculate the fixation index for the groups and subgroups (*f*) according to the estimates of the heterozygosities per locus.

### Private alleles and core collection

To identify plants that represent the genetic diversity of Bahian cacao, two approaches were evaluated: private alleles and core collection. GenAlEx v. 6.5 software was used to detect the private alleles in the Bahian cacao group (based on the STRUCTURE results), subgroups (farm genotypes, SIAL and SIC clones) and modern clones. The same groups and subgroups were used to obtain the core collection, which was identified using 99 iterations in COREFINDER software [30]. This analysis was performed in two steps: in the first step, both the cacao plants of Bahian origin (determined using STRUCTURE) and modern clones were included, and in the second step, only cacao plants of Bahian origin were used.

### Ethics Statement

We confirm that no specific permits were required for the described field studies. The collects were carried out on two research institutions (Cocoa Research Center, Executive Committee of the Cocoa Plantation Plan and Bahian Federal Institute of Education, Science and Technology) and seven private farms, and no specific permissions were required for these locations and activities. We confirm that the owner of the farms gave us permission to conduct the study on these sites. This work was a collaborative study developed by researchers from the Cocoa Research Center, Executive Committee of the Cocoa Plantation Plan (CEPEC, CEPLAC, BA, Brazil), São Paulo's Agency for Agribusiness Technology (APTA, SP, Brazil), Bahian Federal Institute of Education, Science and Technology (IFBAIANO, BA, Brazil), State University of Santa Cruz (UESC, BA, Brazil), State University of Southwest Bahia (UESB, BA, Brazil) and University of Campinas (UNICAMP, SP, Brazil). Also, we confirm that this manuscript is a result of a basic research project, developed mainly at the university and mostly funded by public funding agencies, whose aim is to develop new knowledge and, where the generated results should be shared with the scientific and technological community through open university thesis and manuscripts in conventional scientific journals. In this sense, we would like to state that we fully adhere to all the PLOS ONE policies on sharing data and materials.

We confirm that this study did not involve endangered or protected species.

## Results

### Evaluation of genetic relationships

**Fig 3:**
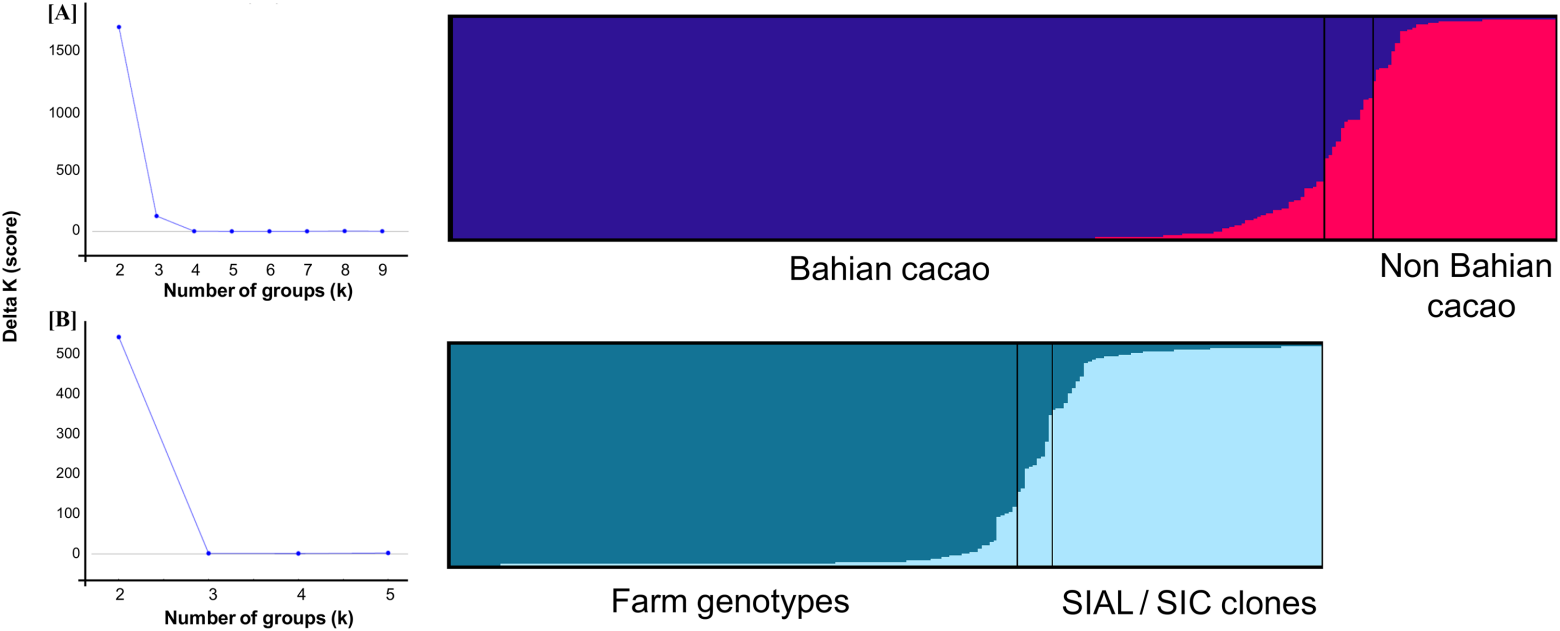
Genetic structure of the cacao clones and genotypes based on Bayesian analyses. The most probable number of groups (K) was estimated based on the method described by Evanno et al. [26]. The colors used in the histograms represent the most likely ancestry of the cluster from which the plants were derived. (A) Analysis performed with 279 cacao plants. (B) Analysis including only the plants identified as belonging to the Bahian cacao group (blue).

Bayesian analyses of the genetic structures of the 279 genotypes and clones indicate the highest ΔK value for K = 2 and a low ΔK value for K = 3 (Fig 3). In this study, the plants were arbitrarily sorted into a particular cluster if they shared at least 70% identity (Q value). Therefore, plants with Q values lower than 70% for a given cluster were treated as having mixed ancestry. For K = 2, the consensus of the 20 Structure runs revealed a large, homogenous cluster of 221 individuals consisting of 168 genotypes collected on Bahian farms, 51 SIAL and SIC clones, and two additional clones, EEG 29 and CAB 14. Because most of the genotypes and clones in this cluster are from Bahia and the cluster has high homogeneity, these plants were considered descendants of the first genotypes introduced into this state. This cluster will hereafter be referred to as the Bahian cacao cluster (BC).

In addition to the BC cluster, a smaller cluster with 46 individuals was observed (Fig 3). This group (denoted the non-Bahian cacao cluster (NBC)) contained 44 clones, which included seven modern cultivars. Two genotypes of Bahian varieties collected on farms were clustered in this group, indicating that they were not of Bahian origin. Twelve plants presented mixed ancestry. For K = 3, a substructure of the BC cluster was observed, and it was subdivided into two groups that separated the Bahian cacao genotypes collected on farms from the Bahian clones (SIAL and SIC). This result was reproduced in the STRUCTURE run performed with only the BC plants (Fig 3), which indicates that a degree of genetic divergence occurred between the descendants of the first cacao plants in Bahia derived from germplasm banks and those that are currently planted on Bahian farms.

The levels of structure among the BC subgroups and modern clones inferred by the G_ST_ statistics are presented in Table 1. All of the pairwise values were significant based on 999 random permutations and 1,000 bootstraps (p ≤ 0.001). The G_ST_ values were higher than 0.2 for almost all of the pairwise evaluations except for those between the SIAL and SIC plants, in which G_ST_ = 0.149. High values were observed between the SIAL and modern clones and between the SIAL clones and farm genotypes, which both had G_ST_ = 0.273.

**Table 1:** Triangular matrix of G_ST_ test results among all cacao plants (168 farm genotypes, 20 SIAL clones, 31 SIC clones and 11 modern clones).

	Farm	SIAL	SIC	Modern
**Farm**	--			
**SIAL**	0.273	--		
**SIC**	0.204	0.149	--	
**Modern**	0.248	0.273	0.224	--

**Fig 4:**
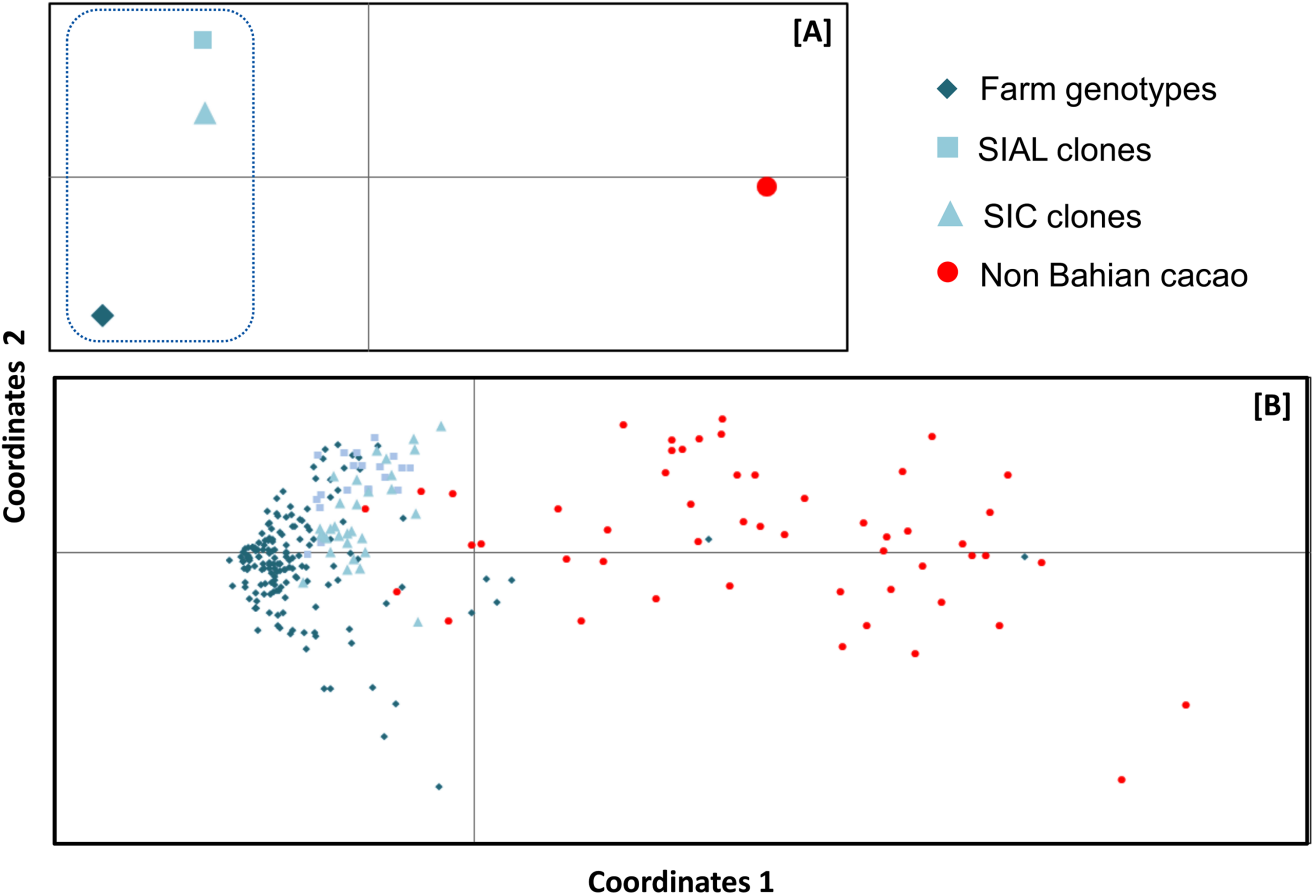
Bidimensional diagram based on a principal coordinate analysis (PCoA) of genotypic data from 30 microsatellite loci. (A) PCoA for cacao groups and (B) PCoA for cacao plants. The dashed line differentiates the groups that are considered Bahian cacao, and the colors used to represent the groups and plants are based on the results from the STRUCTURE software analysis.

The genetic structure obtained from the PCoA of the global data is shown in Fig 4, and it indicates a grouping tendency among the Bahian cacao samples from both farms and germplasm (SIC and SIAL), which corroborates the common origin of these samples suggested by the STRUCTURE results. The CAB 14, EEG 29 and BE 10 clones were placed near the SIAL and SIC clones, and the latter clone was considered to be of mixed ancestry based on the first STRUCTURE run. The modern clones PH 09 and CEPEC 2002 were placed near the plants collected on Bahian farms.

Compared with the NBC plants, the BC plant grouping has less variability. In addition, despite the close proximity between the SIAL and SIC clones and plants obtained from the farms, these subgroups of Bahian plants do not fully match, i.e., the dispersion region of the SIAL and SIC clones is narrower than that of the farm plants.

### Genetic diversity analyses

The polymorphisms in the total data were assessed, and the results are shown in Table 2 and S4 Table. All 30 simple sequence repeat (SSR) loci were polymorphic in the 279 genotypes and clones, and they all produced scorable bands. The analysis revealed a total of 209 alleles and a mean of seven alleles per locus, with a low of two alleles for the mTc-UNICAMP11 locus and high of 16 alleles for the mTc-UNICAMP04 locus. Almost all loci had small observed (H_O_) and expected (H_E_) heterozygosity values, with averages of 0.17 and 0.30, respectively. The PIC values indicated that the mTc-UNICAMP03 locus was the most informative (PIC = 0.62) because the mean of the 30 loci was 0.28. Deviation from Hardy-Weinberg equilibrium was not observed for the mTc-UNICAMP10, mTc-UNICAMP11 and msEstTsh-1 loci (p < 0.01).

**Table 2:** Mean allelic diversity in cacao groups and subgroups at 30 microsatellite loci.

Group/Subgroup	N	N_Ta_	R_a_	N_a_	H_O_	H_E_	Fixation Index
**Total plants**	279	209	2–16	6.97	0.17	0.30	0.36
Bahian cacao	219	113	1–10	3.77	0.08	0.18	0.56
Non-Bahian cacao	46	198	1–16	6.60	0.54	0.62	0.13
**Bahian cacao subgroups**							
Farm cacao	168	86	1–6	2.87	0.07	0.14	0.54
SIAL	20	63	1–7	2.10	0.11	0.16	0.28
SIC	31	71	1–7	2.37	0.12	0.22	0.48
**Modern cultivars**	11	120	2–7	4.00	0.52	0.52	-0.01

*N*, number of plants analyzed; *N*_*Ta*_, total number of alleles; *R*_*a*,_ minimum and maximum alleles per locus; *Na*, mean number of alleles per locus; *H*_*O*_, observed heterozygosity; and *H*_*E*_, expected heterozygosity. Fixation index values are represented by *F* for total plants and *f* for the other groups and subgroups.

The groups formed from the results of the first STRUCTURE run were further evaluated for their genetic diversity statistics (Table 2). For the BC group, the two clones EEG 29 and CAB 14 that were not selected from Bahian farms or from SIC or SIAL germplasm were disregarded. The results showed that the small polymorphism values in the total data were largely influenced by the 219 Bahian cacao plants. Consequently, the diversity parameters for the BC group were low (total alleles = 113; mean number of alleles = 3.77, H_O_ = 0.08 and H_E_ = 0.18) compared with those of the NBC group, which contained 46 plants (total alleles = 198; mean number of alleles = 6.60, H_O_ = 0.54 and H_E_ = 0.62).

Considering their places of origin (farms, SIAL and SIC), the BC subgroups supported the results observed for all plants of Bahian origin (Table 2). Each of these subgroups had low levels of heterozygosity, and lower values were observed for farm plants (with H_O_ and H_E_ of 0.07 and 0.14, respectively). The SIC plants had the highest diversity values among the Bahian subgroups (H_O_ = 0.12 and H_E_ = 0.22). However, the mean number of alleles was slightly higher for the farm subgroup (2.87), followed by the SIC (2.37) and SIAL (2.10) subgroups (Table 2). It is notable that of the 30 loci analyzed in the BC group, three were monomorphic. This observation was corroborated in the subgroups, with 5, 13 and 9 monomorphic loci observed for the farm, SIAL and SIC subgroups, respectively. Because monomorphic loci were not observed in the NBC group, this result again suggests low diversity among the Bahian plants.

To determine the diversity of the cacao that is recommended for planting in Bahia, genetic diversity analyses were performed using only the modern clones. Compared with the BC group, these clones exhibited high diversity (total alleles = 120; mean number of alleles = 4.0, H_O_ = 0.52 and H_E_ = 0.52), and monomorphic loci were not observed (Table 2).

Imbalances for heterozygotes were detected using the fixation index parameters (Table 2). The positive mean *F* value (0.36) indicated a deficiency of heterozygotes in the total analyzed data, with high values observed for the BC group (*f* = 0.56) and its subgroups (farm cacao, *f* = 0.54; SIAL, *f* = 0.28; SIC, *f* = 0.48) and lower values observed for the NBC group (*f* = 0.13) and for the modern clones (*f* = -0.01) (Table 2).

### Private alleles and core collection

The private alleles and core collection from the BC groups, subgroups and modern clones were evaluated. Seventy-one private alleles were obtained from 230 plants (219 Bahian cacao plants from farms, SIAL and SIC and 11 modern cultivars). Forty-four of these alleles were present in the modern cultivars. When the evaluation was performed on the Bahian cacao subgroups, the numbers remained the same, with 13 private alleles observed in farm plants and 5 and 9 private alleles observed in the SIAL and SIC plants, respectively.

**Fig 5:**
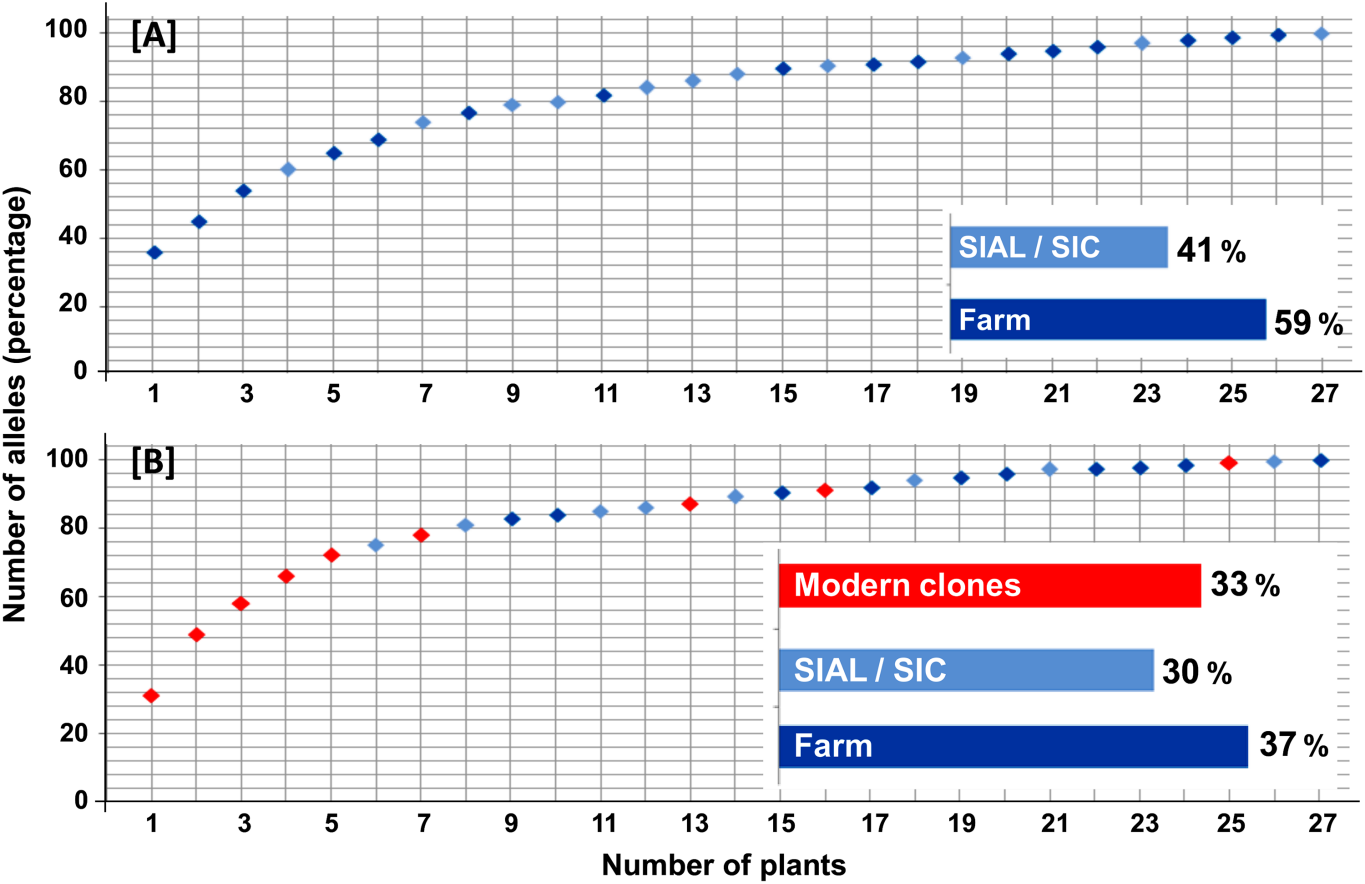
Allelic diversity as a function of the number of plants included in the core collections. (A) Core collection from Bahian cacao plants and (B) Bahian cacao and modern clones. The colors used to represent the groups and plants are based on results from the STRUCTURE software analysis. The number of plants per group composing the core collection is expressed as a percentage.

The diversity of alleles in the modern cultivars and Bahian farm plants was also analyzed using a core collection approach (Fig 5). Regardless of the combination or no of modern Bahian cacao cultivars, 27 plants formed the core collection. In the first analysis, more than 70% of the 160 alleles were present in the first five plants, which were all modern cultivars (CCN 51, PS 1030, PS 1319, SJ 02 and CEPEC 2004). These clones were distributed at different points in the PCoA in quadrants that did not contain Bahian cacao plants, confirming the large genetic distance between these plants (Fig 4).

An analysis of only the Bahian cacao subgroups also indicated a higher number of private alleles (35) in plants from farms, with 5 private alleles observed in the SIAL plants and 12 observed in the SIC plants. In the core collection, more than 70% of the 113 alleles present in Bahian cacao were found in seven plants, and five of these plants were obtained on farms. Once again, the number of plants representing the diversity of Bahian cacao was higher for the farm genotypes (16 plants) than among the SIC (8) and SIAL (3) clones.

## Discussion

Cacao seeds were introduced into Bahia, Brazil in the middle of the eighteenth century, and the descendants of these initial introductions were used to establish nearly all of the commercial cacao farms in Bahia as well as farms in several African countries [10]. The success of these plants in cacao production is reflected in the status of Brazil, Ghana and Nigeria, which are among largest cacao producing countries worldwide [16]. Witches’ broom disease is an important factor that limited production and contributed to Brazil’s drop from the second to sixth largest producer of cacao. Similarly, cacao swollen shoot disease caused significant losses in cacao production in the 1930s in West African countries. The low genetic diversity of Bahian cacao plants observed in this work and previously reported for plants from Bahia cultivars grown in Africa [7] is a potential factor explaining the high susceptibility of these plants to certain diseases. Determining the genetic variability and understanding how it is organized is essential to establishing intervention strategies that promote more efficacious use of available genetic material. This is the first study to characterize Bahian cacao plants derived from germplasm and farms using codominant markers.

### Structure and molecular diversity

The set of microsatellites used in this work effectively assessed the diversity and structure of cacao plants and was used to reconstruct the history of Bahian cacao. The Bayesian approach showed that Bahian cacao forms a homogeneous group, confirming the common origin of the local genotypes currently planted in the state and SIAL and SIC clones, which are used as representatives of Bahian cacao in germplasm banks [11,12].

Although previous studies using phenotypic characteristics and molecular data have suggested that BC is genetically diverse [8,31,32], we did not observe such diversity using a set of 30 highly polymorphic markers. The high homozygosity and homogeneity, which were shown in this study by the lower H_E_ values and higher positive fixation index values, can be explained by the origin of the introduced seeds, dissemination of cacao in Bahia, and reproductive system of cacao. The first cacao seeds to be introduced were most likely collected from descendants of the traditional cultivar Amelonado. Based on molecular markers, low genetic diversity has been reported for this group [33–35] as well as SIAL and SIC clones using dominant markers [36]. In a molecular analysis performed by Aikpokpodium et al. [7] of cultivated and parental plants used for breeding, high fixation index and homogeneity values were observed in Amelonado cultivated in Africa. These plants are descended from Bahian cacao; therefore, these plants share a similar narrow genetic base.

Other traditional varieties, such as Criollo and Nacional, also possess low genetic diversity [37–39]. These clones shared a similar domestication process marked by genetic bottlenecks caused by humans demand for the nutritional benefits of cacao [40]. Amelonado cacao is thought to originate from the Lower Amazon, a region that is not clearly delimited, although it was originally bounded by Marajó Island and the Maicuru River, a tributary of the Amazon River in Pará, Brazil [8]. The origin of the wild plants in the Lower Amazon is uncertain; however, certain populations may have originated from the limited introduction of plants from the Upper Amazon [41], an area considered a center of diversity for cacao plants [31,42], although it remains to be clearly delimited [8]. In addition to the narrow genetic base of the Amelonado-derived populations, the origin of wild plants could explain the genetic similarity between the Upper and Lower Amazon plants reported by Sereno et al. [41] in populations from the Brazilian Amazon.

The mating system of Amelonado cacao may also have influenced the genetic parameters observed in this study. Similar to other traditional cultivars, Amelonado is self-compatible, which may enhance inbreeding. Such inbreeding may be reflected in the positive high fixation index (*f*) values detected in the BC group and subgroups (Table 2), and it has also been reported in other studies of Lower Amazonian cacao [7,41,43]. Additionally, the narrow genetic base of the BC group has been influenced by the introduction of a small number of cacao genotypes in Bahia that have increased endogamy. Therefore, nearly all of the Bahian cacao plants grown over the last 200 years are descended from a limited number of seeds introduced in the middle of the eighteenth and nineteenth centuries. The positive mean fixation index of the NBC plants may be explained by the Wahlund effect, which indicates that the clones originated from different populations with different allele frequencies, thus reducing the overall heterozygosity and consequently increasing the fixation index.

The distribution of the plants in the PCoA corroborates the results obtained from the STRUCTURE software. The presence of clones EEG 29, CAB 14 and BE 10 near the Bahian plants reflects the Lower Amazonian origin of Bahia plants and is consistent with the characterizations in previous studies [31,41]. CAB 14 and BE 10 are wild clones, and although the EEG 29 plants underwent an artificial selection process in a cacao breeding program, they were originally introduced in Bahia; therefore, they share a similar genetic background with Bahian cacao.

Notably, the results of the STRUCTURE, PCoA and Nei’s G_ST_ analyses all corroborate the differences between the Bahian cacao plants collected on farms and those from germplasm banks. The STRUCTURE-based divergence between the farm and clone subgroups could also be observed in the PCoA analysis, in which the genotypes and clones did not completely overlap. The G_ST_ analysis was used to quantify these results. Because of their shared origin in Bahia, the SIAL and SIC clones are expected to be genetically distant from NBC but not from the farm group. Intriguingly, we observed that SIAL and SIC clones showed substantial divergence from both groups (NBC and farm plants) (Table 1), and this result indicates that the clones in these germplasm banks, which are currently considered to be representative of Bahian cacao, do not contain a substantial amount of the total diversity of this group.

The area of the PCoA analysis preferentially contained Bahian cacao clones and did not contain Bahian farm cacao, reflecting the influence of the witches’ broom outbreak on the selection of plants that are more resistant; thus, certain alleles, even those not directly associated with resistance, could have been unknowingly lost.

Additionally, the farm-derived alleles that were not present in the sampled germplasm could be explained by the sampling strategy and objectives of the SIAL and the SIC collections. The SIC clones were reportedly collected from a more restricted area compared with the SIAL clones, with SIC plants collected from an experimental station (currently an educational and research institute) and SIAL plants collected from farms in different municipalities in southern Bahia [11]. The years of seminal propagation on farms and larger collection area used in this work could explain the higher diversity of the group. Moreover, the plants sampled for the SIAL and SIC collections were reportedly chosen for their production characteristics, which explains the genetic separation of these clones from plants on farms because the SIAL and SIC germplasms were not selected to maintain diversity but for use as pioneers in Bahian cacao breeding programs. Based on this information and the results of the different approaches used to assess the genetic structure of Bahia cacao, we recommended the introduction of new materials from farms into the germplasm banks to provide a more effective representation of the diversity of Bahian cacao.

Molecular differences between the SIAL and SIC clones were also detected. Previous studies have indicated that these clones can be effectively differentiated using morphological features, such as pod weight, pod diameter, and wet and dry seed weight [9,11], and higher diversity has been reported among the SIAL clones relative to the SIC clones. Although we detected molecular-level differences between SIAL and SIC clones, the latter showed greater diversity. Therefore, we can conclude that despite their different areas of collection, SIAL clones do not have a broader genetic base than the SIC clones. Using the same set of markers employed in this study, Santos [44] detected the structure between the SIAL and SIC groups, and substructure analyses indicated the formation of three subgroups in the SIC plants, with one formed only of Catongo plants, which is a Comum mutant with white seeds (Fig 2) considered to have good flavor, the subgroup containing Catongo plants have been identified in germplasm collections as SIC clones with numbers greater than 800 [9]. SIC clones identified from 1–100 were selected directly from the experimental area, and clones 100–800 were derived from plants in the first group that self-pollinated. The origin of these SIC clones indicates that they were selected based on productivity and quality; however, this trait combination has not been reported for the selection process of SIAL clones.

### Conservation of diversity and breeding implications

Private alleles and core collections were obtained for the BC plants and combination of BC plants and modern cultivars, and these results provide important information that can be used in conservation programs and breeding strategies for these two groups. Modern clones were selected from Bahia farms starting in 1993 except for CCN 51, which was selected from Ecuador. These selections were based on resistance to witches' broom and productivity [45]. The Bayesian and PCoA results indicated a low genetic relationship among most BC plants and modern clones, thus corroborating previous analyses of genetic distance [44] and indicating the possibility of new combinations between these groups. Therefore, Bahian cacao plants and modern clones that are locally adapted can be used in future breeding programs to increase resistance and productivity and quality characteristics observed in certain varieties of Bahian cacao, such as Maranhão. These improved plants could be tested in Brazil and other countries, including African countries that have been shown to provide favorable environmental conditions for cultivating Amelonado from Bahia. Additional studies should be conducted on the germplasm before breeding, especially before breeding for agronomic traits, and the core collection is a starting point for the selection of plants for evaluation. Such evaluations will allow for the combination of maximal diversity with desirable traits.

Considering only the BC group, the higher occurrence of private alleles in plants from farms relative to the SIAL and SIC subgroups shows that the genotypes from farms possess diversity not found in the clones despite the clones being considered representative of Bahian cacao (Fig 5). In addition to revealing alleles that occur only on farms, this result also corroborates the PCoA result (Fig 4) and diversity parameters obtained for Bahian cacao (Table 2). Therefore, the core collection could be used to select farm plants for sampling, phenotypic evaluations and agronomic evaluations before their addition to germplasm banks to effectively maintain the diversity of Bahian cacao.

When planning a working collection for cacao breeding that represents Bahian cacao, priority should be given to plants with economically useful features (pod and seed size, plant height, productivity, and quality) and maximum genetic diversity. Therefore, the collection should include representatives of the SIC and SIAL cultivars and other types of cacao, such as Comum, Pará, Parazinho, Maranhão, Catongo and Almeida. Because these cultivars have been previously selected by farmers for yield, they could be used by farmers as representatives of a specific type of cacao with a specific feature. For example, white-seeded cacao can be used for Brazilian fine chocolate. Such selections could promote farmer participation in more profitable markets.

### Bahian cacao plants: conclusions and perspectives

Bahian cacao is historically important because it represents the first cacao plants to be cultivated on a large scale in Brazil. Furthermore, to satisfy the growing demand for chocolate drinks in Europe, Bahian Amelonado cacao was first transported in 1822 to African islands, including Príncipe and later São Tomé and Fernando Pó, where it was distributed to several African countries and eventually reached South Asia and Oceania [8,10]. Because of the importance of these plants, African countries became the main cacao producers worldwide, and the cacao plants were known as West African Amelonado [8].

In southern Bahia, the cultivation of cacao deeply influenced the economy, which is reflected in the culture and social patterns of its inhabitants. Not long after the introduction of cacao to Bahia, the seeds had spread along the coastal region, which is now called the cacao coast. Many of the farms cultivated Comum rather than other varieties because this was the first variety to be introduced [5]. Although Bahian cacao is morphologically well defined in the literature because of noticeable differences in the fruits, the analyses performed here could not define the molecular profiles of these plants. Over time, natural and artificial crosses between varieties may have had an effect on their genetic diversity. Indeed, the visual identification of a variety’s typical characteristics was hampered here because many plants possessed fruits that were similar among two or more types.

Notably, certain producers in Bahia allocate a portion of their farms to the production of cacao varieties with no commercial value, and specimens of laranja cacao were found on a farm in the municipality of Gandu. This variety is a mutant of Pará cacao that grows small fruits and seeds (Fig 2). Reports from individuals connected to the farm indicate that this variety is cultivated to preserve the history of Bahian cacao, with the fruits sometimes used for ornamental purposes.

The attitude towards preservation observed in certain farmers is the result of a shift in global perspective that promotes sustainable agriculture. Thus, certain producers in Bahia have been encouraged to open their farms to ecotourism. On such tours, it is possible to contemplate the wonders of the remaining Atlantic Forest, whose leafy trees have been maintained to shade the cacao, and understand how the seeds are cultivated and, in some cases, processed. Additionally, the production of organic and fine cacao may provide additional value, and a number of farmers have considered this alternative production strategy because of the cacao crisis in Bahia, which has been a problem for over two decades. Cacao seeds from Bahia are receiving awards at national and international competitions, attesting to the quality of these materials and indicating the potential of increasing producer's earnings three-fold in the gourmet market. The Maranhão and Catongo varieties are used in the fine cacao market, with Catongo reportedly producing less astringent and more flavorful chocolate [17].

Amelonado cacao, both in Bahia and Africa, is preferred by certain farmer because of its high productivity [7,44]. After two decades of managing witches' broom in Bahia, with research reports indicating the high susceptibility of Bahian cacao to this disease, the high productivity of Bahian cacao trees is surprising, especially after two or more generations in a family. These trees survived the disease’s critical infection period and appear to have a degree of tolerance to it, although at more moderate levels than what is exhibited by modern clones. Additionally, West African Amelonado is less susceptible to black pod, an important disease in African countries. Recent sensorial studies with different types of cacao have indicated that Comum cacao produces a chocolate with acceptable flavor that is sweeter, and the plant produces many cotyledons, which indicates a good production yield when processed [46]. Considering the historic importance of cacao from Bahia for the economic and social structure of countries worldwide, new resistance and productivity analyses will provide valuable information on reactions to disease and provide for new selections aimed at Bahian cacao breeding. After testing, these plants could also be used for planting in other countries with environmental conditions similar to those in Bahia. Based on the molecular data shown here, the inclusion of farm plants in germplasm banks could effectively conserve the genetic background of Bahian cacao.

## Supporting Information

S1 Fig Mutants of Para cacao. (A) Parazinho and (B) Tomate.
(TIFF)

S2 Fig Varieties of Bahian cacao. (A) and (C) ripe and green fruits of Pará variety and, (B) and (D) ripe and green fruits of Comum variety.
(TIFF)

S1 Table Characteristics of the fruits of local Bahian cacao varieties. (XLSX)

S2 Table Origin and number of Bahian cacao genotypes collected on farms in Southern Bahia. (XLSX)

S3 Table List of the 103 cacao clones collected in germplasm banks and farms and their origins. Modern cultivars used in commercial plantations on Bahia farms are indicated by the superscript *a*.
(XLSX)

S4 Table Summary statistics of allelic diversity across 30 microsatellite loci in cacao plants. *Na* number of alleles, *H*_*O*_ observed heterozygosity, *H*_*E*_ expected heterozygosity, *PIC* polymorphism information content, and *F* fixation index. Significance level departs from Hardy-Weinberg equilibrium after Bonferroni correction at ^a^p < 0.003.
(XLSX)
