# Supplementary figures and images for: Genetic Structure and Molecular Diversity of Cacao Plants Established as Local Varieties for More than Two Centuries: The Genetic History of Cacao Plantations in Bahia, Brazil
Source: PLoS One. 2015 Dec 16;10(12):e0145276. doi: 10.1371/journal.pone.0145276 (PMC4682715; doi:10.1371/journal.pone.0145276)

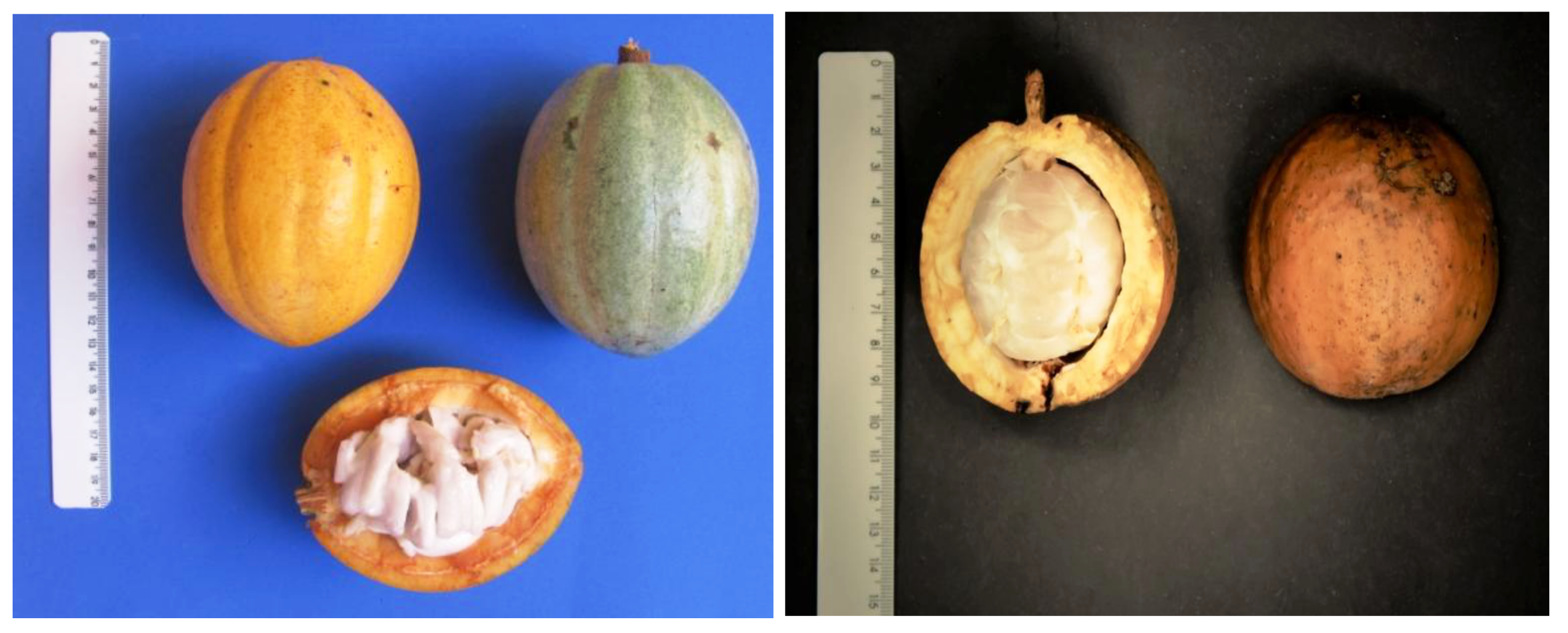

Supplement: S1 Fig — (A) Parazinho and (B) Tomate. (TIFF) [file pone.0145276.s001.tiff]

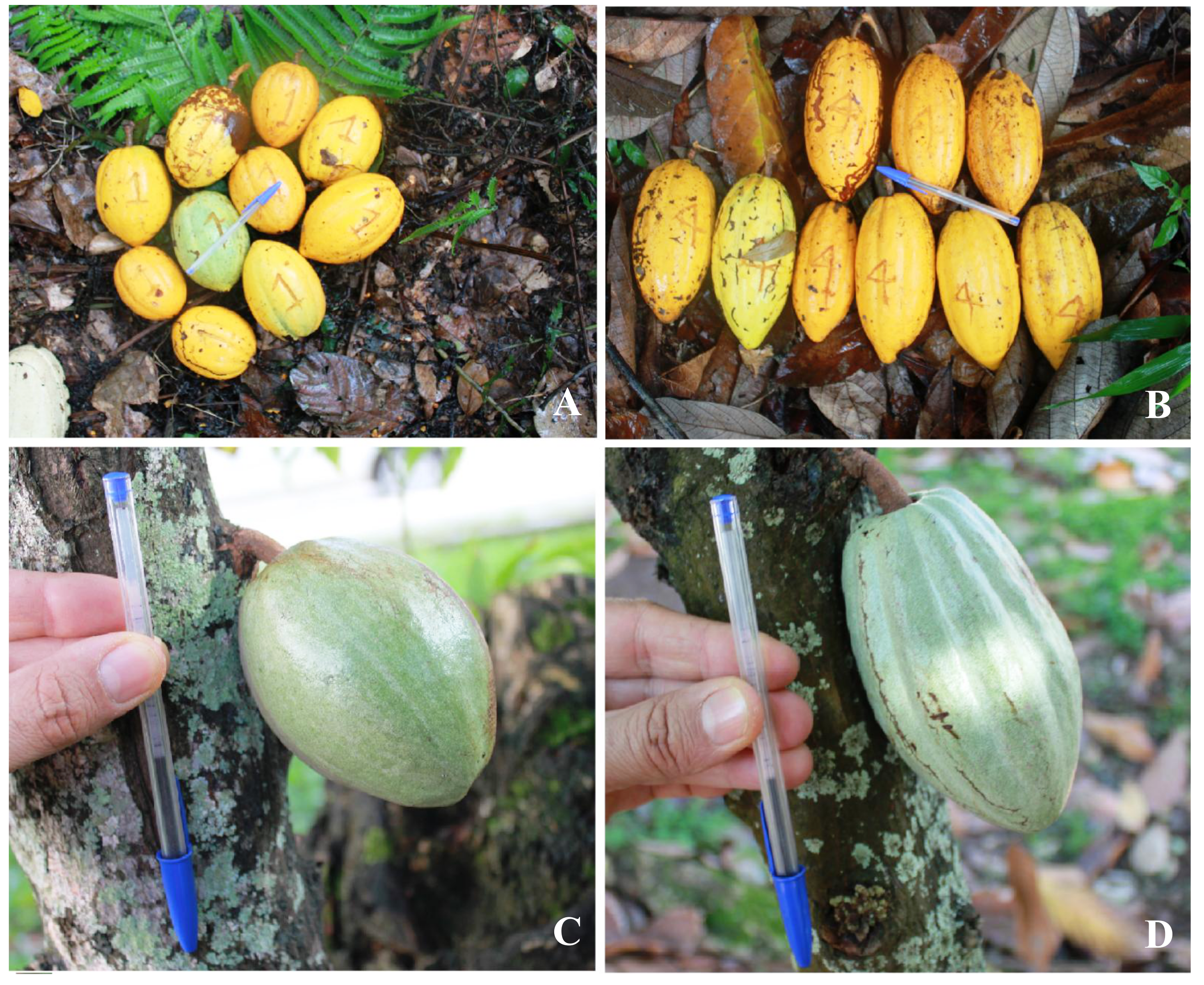

Supplement: S2 Fig — (A) and (C) ripe and green fruits of Pará variety and, (B) and (D) ripe and green fruits of Comum variety. (TIFF) [file pone.0145276.s002.tiff]
